# Clinical Outcomes of Biportal Endoscopic Interlaminar Decompression with Oblique Lumbar Interbody Fusion (OLIF): Comparative Analysis with TLIF

**DOI:** 10.3390/brainsci11050630

**Published:** 2021-05-13

**Authors:** Ho-Jin Lee, Eugene J. Park, Jae-Sung Ahn, Sang Bum Kim, Youk-Sang Kwon, Young-Cheol Park

**Affiliations:** 1 Department of Orthopaedic Surgery, Chungnam National University College of Medicine, Daejeon 35015, Korea; leeleo98@gmail.com (H.-J.L.); sangbumos@me.com (S.B.K.); 2 Department of Orthopaedic Surgery, Kyungpook National University School of Medicine, Daegu 41405, Korea; pjj841229@gmail.com; 3 Department of Orthopaedic Surgery, Daejeon Centum Hospital, Daejeon 35209, Korea; ortho82@gmail.com; 4 Department of Orthopaedic Surgery, Medical Battalion, The 13th Special Mission Brigade, Special Warfare Command, Chungbuk 28644, Korea; ycyeh0830@hanmail.net

**Keywords:** lumbar vertebrae, biportal endoscopy, oblique lateral interbody fusion, minimally invasive surgery, severe central canal stenosis, foraminal stenosis, segmental instability

## Abstract

Oblique lumbar interbody fusion (OLIF) improves the spinal canal, with favorable clinical outcomes. However, it may not be useful for treating concurrent, severe central canal stenosis (SCCS). Therefore, we added biportal endoscopic spinal surgery (BESS) after OLIF, evaluated the combined procedure for one-segment fusion with clinical outcomes, and compared it to open conventional TLIF. Patients were divided into two groups: Group A underwent BESS with OLIF, and Group B were treated via TLIF. The length of hospital stay (LOS), follow-up period, operative time, estimated blood loss (EBL), fusion segment, complications, and clinical outcomes were evaluated. Clinical outcomes were measured using Visual Analog Scale (VAS) scores, Oswestry Disability Index (ODI) scores, and the modified Macnab criteria. All the clinical parameters improved significantly after the operation in Group A. The only significant between-group difference was that the EBL was significantly lower in Group A. At the final follow-up, no clinical parameter differed significantly between the groups. No complications developed in either group. We suggest that our combination technique is a useful, alternative, minimally invasive procedure for the treatment of one-segment lumbar SCCS associated with foraminal stenosis or segmental instability.

## 1. Introduction

Lateral lumbar interbody fusion (LLIF) is a minimally invasive procedure used to treat degenerative spinal diseases and includes extreme lateral interbody fusion (XLIF), direct lateral interbody fusion (DLIF), and oblique lumbar interbody fusion (OLIF) [1,2,3]. LLIF corrects coronal and sagittal deformities via ligamentotaxis and indirectly decompresses the neural canal by restoring disc height, stabilizing segmental instability, and remodeling the spinal canal [2,4,5,6]. Indirect decompression affords various advantages compared to direct decompression, including lower risks of neural injury, incidental durotomy, and postoperative perineural fibrosis [4,7]. OLIF is performed via a corridor located anterior to the psoas muscle; XLIF or DLIF require psoas muscle penetration [1,3]. Thus, OLIF enables cage insertion with less psoas injury and no need for nerve monitoring. It also offers easy access during lumbosacral junction-level surgery. The iliac crest, which poses an obstacle during XLIF or DLIF, is evaded [4].

However, pathological structures in the posterior column, including a hypertrophied ligamentum flavum or facet spurs, cannot be removed via the indirect decompression of OLIF [8]. Nakashima et al. [9] reported that a severe, preoperative, central canal stenosis or the ossification of the posterior longitudinal ligament (OPLL) are contraindications for LLIF. Heo et al. [8,10] suggested that OLIF was not always indicated for patients with severe canal stenosis or concomitant ruptured disc herniation.

Therefore, we combined a technique allowing posterior decompression in patients with severe central canal stenosis (SCCS) to OLIF. To ensure adequate decompression, we added biportal endoscopic spinal surgery (BESS); we performed unilateral laminotomy for bilateral decompression (ULBD) after OLIF. BESS offers many advantages as an endoscopic procedure. It is easy, given the excellent surgical view afforded by the independent working tube [11,12,13], and it is associated with low complication rates [13,14,15,16]. Hence, it is increasingly applied for posterior decompression with minimal invasiveness. We previously described our combination procedure in detail and presented the clinical results [17]. BESS can be used during OLIF to treat SCCS in a minimally invasive manner efficiently. Here, we evaluated this combined procedure with the clinical outcomes for one-segment fusion and compared it to open conventional TLIF.

## 2. Materials and Methods

We complied with the ethical standards of our institutional review board (approval no. CNUH 2019–11–047) and those of the 1964 Helsinki declaration and later amendments, or comparable ethical standards. Since May 2015, we have performed the OLIF procedure on 157 patients and conventional TLIF on 168. Of these, 23 underwent BESS with OLIF and 45 received TLIF on a single segment of the lumbar spine. The excluded patients from the two operations underwent procedures that involved a multi-segment performance, a revision operation, and the OLIF without the BESS decompression procedure. Finally, twenty patients for each procedure with follow-up times that exceeded 12 months were enrolled and retrospectively analyzed. Patients were divided into two groups: Group A (BESS with OLIF) and Group B (TLIF).

### 2.1. Indications for Operations

**Figure 1:**
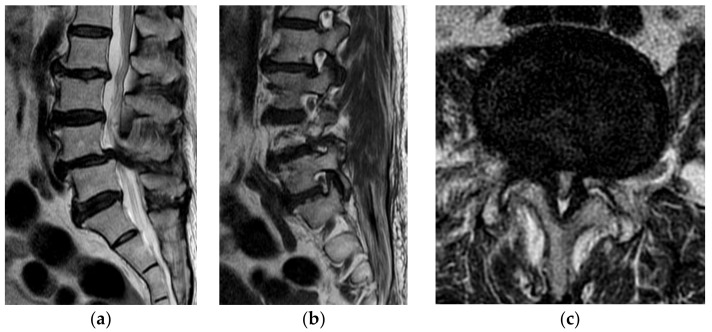
A 71-year-old female presented with severe radiating pain in both legs, with claudication. (**a**) The sagittal MR image shows central canal stenosis with spondylolisthesis at the L4–5 level. (**b**) The right parasagittal MR image shows severe foraminal stenosis. (**c**) An axial MR image shows severe central canal stenosis at the L4–5 level. Facet hypertrophy, thickening of the ligamentum flavum, and the bulging disc severely compress the dural sac.

The indications for the operation were segmental instability or foraminal stenosis contained SCCS (Figure 1). The patients with one-segment SCCS evident in a lumbar spine MRI and associated neurogenic claudication, with or without paresthesia of the lower extremities, who were refractory to conservative treatment were included.

Segmental instability was defined as a translation above 4.5 mm or over 15° of angulation change evident in flexion and extension simple radiographs [18]. Foraminal stenosis was scored using the Wildermuth grading system [19]; moderate to severe cases were included. Central canal stenosis was measured using the Lumbar Central Canal Stenosis (LCCS) grading system [20]; those with severe (grade 3) stenosis were included. The exclusion criteria were scoliosis with a Cobb angle >30° [21], an infection, a tumor, trauma, and revision surgery (Table 1).

### 2.2. Surgical Procedures

All procedures were performed by two board-certified orthopedic spine surgeons (JSA and HJL) working at the same institute.

#### 2.2.1. BESS with OLIF

**Figure 2:**
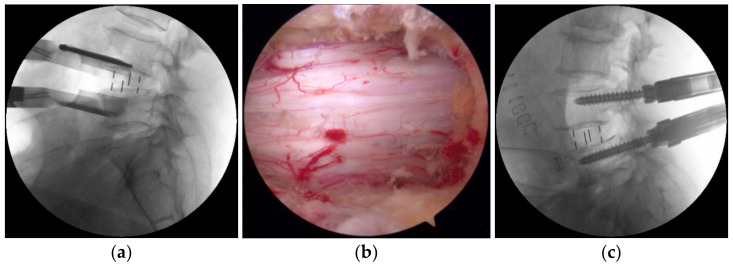
The BESS with OLIF procedure: (**a**) A fluoroscopic image taken during OLIF. (**b**) An endoscopic image showing the thecal sac after decompression. After OLIF, BESS was used to perform ULBD after the patient was changed to the prone position. (**c**) A PPF intraoperative fluoroscopic image. PPF was used for posterior fixation after ULBD of BESS. BESS, biportal endoscopic spinal surgery; OLIF, oblique lumbar interbody fusion; ULBD, unilateral laminotomy for bilateral decompression; PPF, percutaneous pedicle screw fixation.

After the induction of general anesthesia, each patient was placed in a true lateral decubitus position with the right side down on a radiolucent operating table. Each patient initially underwent OLIF via the retroperitoneal approach; this featured abdominal wall muscle separation, the application of a tubular retractor, a discectomy, endplate preparation, and cage insertion. Polyether-ether-ketone (PEEK) cages filled with a demineralized bone matrix (DBM) (Grafton; Medtronic, Minneapolis, MN, USA) were inserted. If surgery on segments L2 to L5 was required, OLIF25 was performed as described previously [3,4,6], and a Clydesdale PEEK cage (Medtronic Sofamor Danek, Memphis, TN, USA) was inserted (Figure 2a). To prevent over-distraction while inserting the cage, we measured the average height of the adjacent segments’ disc space preoperatively to determine the adequate height of the operative level. We performed OLIF51 to treat the L5-S1 segment. The L5-S1 level was identified in C-arm lateral images, and a transverse skin incision was created between the pubic symphysis and the umbilicus. After sequential abdominal muscle splitting/dissection and retroperitoneal fat exposure, the peritoneum was retracted medially. The left common iliac artery and vein were identified and retracted laterally to expose the L5-S1 disc space. The middle sacral artery and vein were ligated prior to the annulotomy. After total discectomy and endplate preparation, a Perimeter PEEK cage (Medtronic Sofamor Danek, Memphis, TN, USA) was inserted.

**Figure 3:**
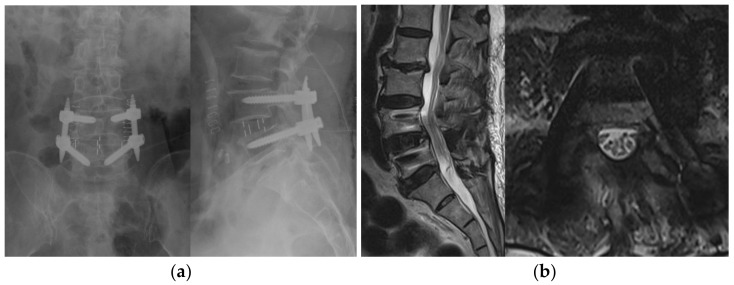
(**a**) A postoperative, simple radiographic image after BESS with OLIF. (**b**) The postoperative MR image reveals the complete decompression of SCCS at L4–5. BESS, biportal endoscopic spinal surgery; OLIF, oblique lumbar interbody fusion; SCCS, severe central canal stenosis.

After OLIF, the patient was changed to the prone position. Additional posterior decompression was performed using the interlaminar approach of BESS [14]. After creating two separate skin incisions above and below the margin of the interlaminar space, the left incision usually served as a viewing portal and the right incision as a working portal. ULBD was achieved under endoscopic guidance (Figure 2b). The adequate decompression was confirmed when the bilateral borders of the thecal sac were exposed with the free movement of the traversing nerve root during ULBD. After BESS decompression, a percutaneous pedicle screw system (CD Horizon Longitude II; Medtronic Sofamor Danek, Memphis, TN, USA) was used for posterior fixation (Figure 2c and Figure 3).

#### 2.2.2. Conventional Open TLIF

After the induction of general anesthesia, the patients were placed prone on a radiolucent operating table. All underwent conventional, open, one-segment TLIF, as described previously [22,23]. A Capstone PEEK cage (Medtronic Sofamor Danek, Memphis, TN, USA) packed with autograft material derived via facetectomy and DBM (Grafton; Medtronic, Minneapolis, MN, USA) was inserted. An open pedicle screw system (Xia 3; Stryker, Allendale, NJ, USA) was used for posterior fixation.

### 2.3. Clinical Assessment

Patients were preoperatively stratified using the American Society of Anesthesiologist Physical Status Index. The length of stay (LOS), follow-up period, operative time, estimated blood loss (EBL), fusion segment, complications, and clinical outcomes were recorded. Within Group A, Visual Analog Scale (VAS) scores and the Oswestry Disability Index (ODI) scores were recorded preoperatively; at 1, 3, 6, and 12 months; and at the final follow-up postoperatively for a clinical outcomes analysis. The modified Macnab criteria were recorded at the final follow-up. For all patients who underwent either BESS with OLIF or conventional open TLIF, the clinical scores were recorded preoperatively and during the follow-up, as were the clinical outcomes at the final follow-up at least one year after surgery.

### 2.4. Statistical Analyses

IBM SPSS version 22 software (IBM Corp., Armonk, NY, USA) was used for all statistical analyses. The normality of the data distribution was checked using the Kolmogorov–Smirnov test. We present means ± standard deviations (SDs) for data with normal distributions. Otherwise, we present medians with ranges. The Friedman test was used for the intragroup comparisons of clinical outcomes within Group A. Postoperative and preoperative data were compared after the application of the Bonferroni correction. The demographic data, operative details, and clinical outcomes of the two groups were compared using the Student *t*-test for normally distributed data and the Mann–Whitney U-test otherwise. The chi-square test or the Fisher exact test were used to compare categorical variables. A *p*-value < 0.05 was considered to reflect statistical significance.

## 3. Results

### 3.1. Demographic Data, Disease Characteristics, and Operative Data

Baseline patient demographics, disease characteristics, and operative data are summarized in Table 2.

In all, 40 patients were enrolled, and each group consisted of 20 patients. Foraminal stenosis with SCCS was the principal diagnosis in both groups (60% of Group A and 55% of Group B). No significant between-group differences were evident either demographically or in terms of disease characteristics. The mean follow-up periods were 17.6 ± 5.6 months for Group A and 19.3 ± 4.5 months for Group B; the difference was not significant. There were no between-group differences in any operative parameter, including the ASA classification, LOS, and operative time. The mean EBL was significantly lower in Group A (151 ± 60.9 mL) than Group B (435 ± 243.0 mL) (*p* < 0.05). L4–5 was the most frequently involved fusion segment in both groups. No complications, including dural tearing, postoperative neuropathy, or infection, were recorded in either group.

### 3.2. Clinical Outcomes

#### 3.2.1. Intra-Group Analyses (Group A)

**Figure 4:**
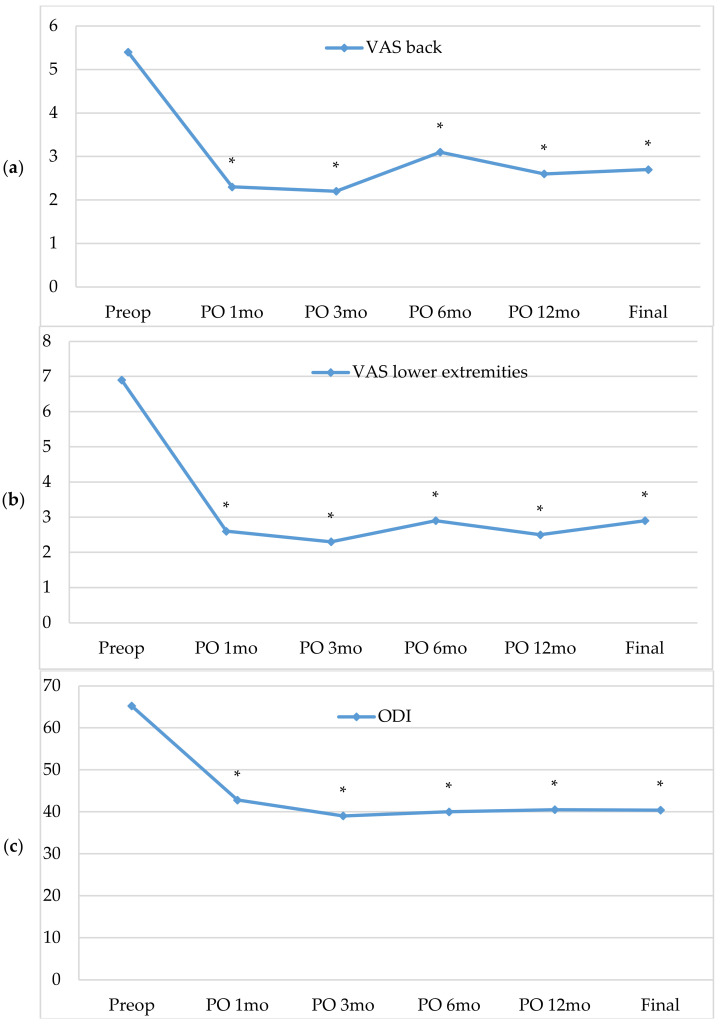
Clinical outcomes of Group A. (**a**) VAS back scores. (**b**) VAS lower extremity scores. (**c**) ODI scores. VAS: visual analog scale, ODI: Oswestry Disability Index; PREOP: preoperative value; PO: postoperative value. * *p*-value < 0.05 compared to the preoperative value.

The clinical outcomes of Group A, including the VAS scores of the back and lower extremities and the ODI, are listed in Table 3. All follow-up clinical parameters improved significantly compared to their preoperative status (Figure 4). In terms of the modified Macnab criteria, good to excellent results (a satisfied outcome) were evident in 85% of patients.

#### 3.2.2. Inter-Group Analyses (Group A Versus Group B)

The clinical outcomes of the two groups are summarized in Table 4. The preoperative ODI scores were 65.2 ± 15.2 and 51.9 ± 16.9, respectively, and were significantly higher in Group A (*p* = 0.01). However, the final ODI scores of the two groups did not differ significantly. Moreover, the final follow-up VAS scores for the back and lower extremities, and the modified Macnab criteria, did not differ significantly between the two groups.

## 4. Discussion

The use of BESS combined with OLIF to treat patients with one-segment problems improved the clinical outcomes, including the back and lower-extremity VAS scores, the ODI score, and the modified Macnab criteria, without any complications (Table 2, Table 3 and Table 4). We recorded exceptional symptomatic and functional improvements in our 20 patients with SCCS. Moreover, the combination procedure did not differ significantly from conventional open TLIF in terms of any operative parameter or clinical outcome analyzed, except the EBL (Table 2 and Table 4). The EBL of Group A was much lower than that of Group B; the difference was statistically significant. We found that BESS with OLIF was a useful alternative treatment for one-segment fusion cases with SCCS.

OLIF featuring an oblique corridor has recently been used to reduce complications associated with XLIF and DLIF, including possible lumbar plexus injury, psoas muscle injury attributable to use of the transpsoas approach, and anatomical obstacles encountered during the approach on the level of the lumbosacral junction [3,4,24,25,26]. OLIF is associated with fewer approach-related complications and can significantly improve the spinal canal both axially and foraminally via indirect decompression [4,6]. However, it does not adequately treat some cases of lumbar degenerative disease. Oliveira et al. [5] reported that severe central canal stenosis combined with lateral or foraminal stenosis attributable to osteophyte formation was a contraindication for LLIF; in that study, the additional decompression of such lesions was required given the persistence of stenotic symptoms. Nakashima et al. [9], in a prospective cohort study, concluded that preoperative SCCS or OPLL was a contraindication for LLIF, and suggested that indirect lumbar decompression using LLIF should not be considered for SCCS or OPLL patients.

Heo et al. [8,10] reported that OLIF alone did not adequately treat severe canal stenosis or concurrent ruptured disc herniation. They added endoscopic discectomy to OLIF before inserting the fusion cage (from the anterior) and found that concomitant central or foraminal herniated discs were completely removed, and the method was a useful, alternative, minimally invasive surgical option. However, they also reported that the technique has certain limitations when used to treat stenosis caused by facet hypertrophy, the thickening of the ligamentum flavum, or calcified disc herniation, and that a posterior approach should be used for such cases. Previously, we combined BESS with OLIF (a minimally invasive combination technique) and described the clinical outcomes [17]. BESS decompression was performed in the manner of ULBD, thus through two independent portals. We removed the central canal stenotic lesions, including hypertrophied facets, the ligamentum flavum, and herniated discs, via small incisions. Our patients reported significant symptom relief [17].

To perform adequate decompression using BESS after the OLIF procedure, previous experience with ULBD using BESS is necessary. Because interlaminar decompression after OLIF is typically performed in a lumbar extension posture to make lordosis, it is more technically demanding due to the narrowed interlaminar space. In our cases, BESS decompression after OLIF was performed by an expert biportal endoscopic spine surgeon.

We used our combination procedure to treat patients with one-segment SCCS and either foraminal stenosis or segmental instability. In our experience, BESS with OLIF affords many advantages. First, the indications for OLIF can be extended if BESS is applied. As described above, OLIF alone does not adequately treat some degenerative spine diseases given the lack of direct decompression. We assumed that large cage insertion would not fully decompress SCCS. Remnant stenosis was evident in the follow-up MRI after the initial surgery on multi-segment OLIF cases who underwent staged operations with a 1 week interval [17]. Thus, BESS can be used to additionally perform ULBD, complementing the OLIF deficit in a minimally invasive manner. The mean operative time required for additional endoscopic decompression was only 50 min; we encountered no complications. Second, our procedure significantly reduces bleeding compared to open conventional TLIF (Table 2). We did not use any specific perioperative blood conservation strategies for both groups, such as the use of antifibrinolytics, intraoperative controlled hypotension, or intraoperative cell salvage. The mean EBL was approximately 150 mL, or 35% of that of conventional TLIF. Our combination procedure is appropriate for older patients with comorbidities or of advanced ASA grade. Third, BESS with OLIF may minimize posterior myo-ligamentous injury; this is considered a drawback of TLIF and PLIF. Our procedure requires less retraction of the paraspinal muscles than other methods that use a microscope or tubular retractors; muscle atrophy is minimized. In a previous study, reversible changes in back muscles after BESS were noted in the postoperative follow-up MRI [27]. BESS allows meticulous decompression and minimal soft tissue manipulation because the procedure is performed under a maximum magnification of 28:1–35:1 and a brightness of 2700–6700 lux [28]. Fourth, the procedure time of the combination method is not excessive at 182 min, and is thus longer than TLIF. However, this includes the time taken to change the patient from the lateral to the prone position, and surgical draping; the difference is not statistically significant (Table 2).

The present study had certain limitations. The most important are our small number of cases and the relatively short follow-up. To compensate for these limitations, we analyzed all patients in two different ways, namely, in an intra-group and inter-group manner, with a comparable control group. In intra-group analyses, we evaluated the postoperative clinical improvements (compared to preoperative status) after the use of our combination technique. In inter-group analyses, we objectively compared our technique to the well-proven traditional procedure. Another limitation was the lack of radiological data. Prospective cases undergoing longer-term follow-up both clinically and radiologically are required for the further evaluation of BESS with OLIF.

## 5. Conclusions

We found that this combination procedure did not differ significantly from conventional open TLIF in terms of any operative parameter or clinical outcome analyzed, except the EBL. The addition of BESS to OLIF complemented the deficit of OLIF in a minimally invasive manner and enhanced the inherent advantages of OLIF as an advanced technique. We suggest that our combination technique is a useful, alternative, minimally invasive procedure for the treatment of one-segment lumbar SCCS combined with foraminal stenosis or segmental instability.

## Figures and Tables

**Table 1:** Inclusion and exclusion criteria.

Inclusion Criteria (All of 1–3):
1. Lower back pain and/or leg pain with neurogenic intermittent claudication (NIC) and a progressive neurological deficit with:
- segmental instability
>4.5 mm of translation or 15° of angulation evident on a flexion-extension radiograph [18],
or
- foraminal stenosis
moderate to severe based on the Wildermuth grading system [19].
2. Concomitant, severe central canal stenosis
based on the Lumbar central canal stenosis (LCCS) grading system [20].
3. Failure of 3 months of conservative treatment.
Exclusion criteria (any of 1–4):
1. Scoliosis with a Cobb angle > 30° [21]
2. Coexisting pathological conditions
- infection,- tumor
3. Trauma
4. Revision surgery

**Table 2:** Demographic data, disease characteristics, and operative data.

	Group A	Group B	*p*-Value
Patients, no.	20	20	
Mean age ± SD, years	68.4 ± 5.6	66.5 ± 6.8	0.34
Male/female ratio	9:11	8:12	1.0
Diagnosis (SCCS with), no. (%)			
Foraminal stenosis	12 (60)	11 (55)	0.83
Segmental instability	3 (15)	5 (25)	
Foraminal stenosis + segmental instability	5 (25)	4 (20)	
ASA classification ± SD, grade	2.4 ± 0.5	2.2 ± 0.4	0.15
Median LOS, days (range)	14 (11–17)	14 (13–18)	0.48
Mean FU ± SD, months	17.6 ± 5.6	19.3 ± 4.5	0.30
Mean operative time ± SD, min	182 ± 42.9	167 ± 21.2	0.15
Mean EBL ± SD*mL	151 ± 60.9	435 ± 243.0	0.00
Fusion segment, no. (%)			
L2–3	1	0	0.74
L3–4	2	4	
L4–5	12 (60)	10 (50)	
L5-S1	5	6	
Complications, no.	0	0	

SCCS: severe central canal stenosis, ASA: American Society of Anesthesiologists, LOS: length of stay, FU: follow-up, EBL: estimated blood loss, * *p* < 0.05.

**Table 3:** Clinical outcomes of Group A (the values are means ± standard deviations).

	Group A	*p*-Value (Compared to the Preoperative Value)
VAS back scores		
Preoperative	5.4 ± 2.4	
1 month postoperative	2.3 ± 2.0	0.000
3 months postoperative	2.2 ± 2.1	0.000
6 months postoperative	3.1 ± 2.2	0.030
12 months postoperative	2.6 ± 2.1	0.002
Final FU	2.7 ± 2.2	0.003
VAS lower extremity scores		
Preoperative	6.9 ± 2.1	
1 month postoperative	2.6 ± 2.5	0.000
3 months postoperative	2.3 ± 1.8	0.000
6 months postoperative	2.9 ± 2.3	0.001
12 months postoperative	2.5 ± 1.9	0.000
Final FU	2.9 ± 2.0	0.001
ODI scores		
Preoperative	65.2 ± 15.2	
1 month postoperative	42.8 ± 14.9	0.030
3 months postoperative	39.0 ± 14.5	0.000
6 months postoperative	40.0 ±17.7	0.001
12 months postoperative	40.5 ± 17.6	0.000
Final FU	40.4 ± 16.5	0.001
Modified Macnab criteria (cases, %)		
Excellent	7 (35)	
Good	10 (50)	
Fair	3 (15)	
Poor	0 (0)	

VAS: visual analog scale; FU: follow-up; ODI: Oswestry Disability Index. * *p* < 0.05.

**Table 4:** A comparison of clinical outcomes between Groups A and B (the values are means ± standard deviations).

	Group A	Group B	*p*-Value
VAS back scores			
Preoperative	5.4 ± 2.4	4.7 ± 2.3	0.35
Final FU	2.7 ± 2.2	3.4 ± 2.2	0.36
VAS lower extremity scores			
Preoperative	6.9 ± 2.1	5.8 ± 1.7	0.06
Final FU	2.9 ± 1.9	4.2 ± 2.3	0.07
ODI scores			
Preoperative*	65.2 ± 15.2	51.9 ± 16.9	0.01
Final FU	40.4 ± 16.5	38.2 ± 15.2	0.66
Modified Macnab criteria (cases, %)			
Excellent	7 (35)	3 (15)	0.28
Good	10 (50)	10 (50)	
Fair	3 (15)	6 (30)	
Poor	0 (0)	1 (5)	

VAS: visual analog scale; FU: follow-up; ODI: Oswestry Disability Index, * *p* < 0.05.

## Data Availability

The data presented in this study are available on request from the corresponding author. The data are not publicly available due to the applicable data protection law.
